# Investigation on the effect of vitamin C on growth & biofilm-forming potential of *Streptococcus mutans* isolated from patients with dental caries

**DOI:** 10.1186/s12866-020-01914-4

**Published:** 2020-07-30

**Authors:** Zehdi Eydou, Bader Naser Jad, Zeyad Elsayed, Anas Ismail, Michael Magaogao, Ashfaque Hossain

**Affiliations:** 1 RAK College of Medical Sciences, RAK Medical and Health Sciences University, 11172 Ras Al Khaimah, UAE; 2 Department of Medical Microbiology and Immunology, RAK Medical and Health Sciences University, 11172 Ras Al Khaimah, UAE

**Keywords:** *S. mutans*, Vitamin C, Biofilm, BPC, MIC, MBC

## Abstract

**Background:**

*Streptococcus mutans* is a major cause of dental caries. Its capacity to produce biofilm is fundamental in the pathogenesis of this ubiquitous condition. As maintaining a healthy dentition is a genuine goal given the contemporary advance in caries control, researchers are striving to achieve a breakthrough in caries therapy. We are taking the anti-cariogenic properties of vitamin C a step-further, considering the well-known evidence of the inversely proportionate relationship between salivary levels of vitamin C and dental caries. The aim of this study was to determine MIC, MBC, biofilm prevention concentration (BPC), and derivative measures of vitamin C against fresh clinical isolates of *S. mutans* to evaluate its efficacy as an anti-cariogenic agent.

**Results:**

Based on the data of four independent experiments done in quadruplicates, we found a concentration-dependent inhibitory effect of vitamin C on all *S. mutans* strains tested. The average MBC, MIC, and BPC of vitamin C were found to be 10.16, 9.38, and 5.61 mg/ml, respectively. Spectrophotometric quantitation of crystal violet showed diminished biofilm formation in the presence of vitamin C (*p* < 0.05). When compared with gentamicin, vitamin C produced a zone of inhibition that was three times as large against the clinical isolates.

**Conclusion:**

Our results show that vitamin C has a negative effect on *S. mutans* growth and biofilm formation. Being the first to meticulously utilize BPC to explore a well-known effect of vitamin C, this report aims to help in the instigation of trials of higher evidence that will ultimately culminate in repurposing vitamin C as a novel anti-cariogenic agent, albeit further studies are required to provide auxiliary evidence in this context.

## Background

Dental caries is one of the world’s most common medical conditions, and it goes as far as the World Health Organization estimating that 2.3 billion people suffered from caries of permanent teeth in 2017 [1]. Among others, *Streptococcus mutans*, which is a renowned member of the mutans streptococci group and naturally found in the oral cavity, is broadly accepted to be a major dental caries pathogen [2]. This remains unchallenged in spite of the trending consensus in the oral biology community that favors some form of an ecological-based hypothesis, in which dysbiosis or a general imbalance of acidogens is to be blamed as the etiology of caries rather than inculpating a specific microbial agent [3]. Further validating *S. mutans* role in the multifactorial caries process is the fact that most advocates of the ecological plaque hypothesis still acknowledge some level of specificity or reproducibility to the taxa associated with decayed surfaces [4].

The cariogenic property of *S. mutans* is attributed to a host of factors, imperatively biofilm formation and its antecedent processes [5]. Biofilms are multicellular complex communities of bacteria that can adhere to nearly any surface [6]. On clinical grounds, they are produced to allow the bacteria to survive in response to stresses in the form of antibiotics, nutrient limitation, changes in environmental temperature or oxygen, and others [7]. Biofilms have gained much clinical relevance not only in dentistry but also in medical practice, as they are thought to be responsible for up to two-thirds of hospital-acquired infections [8] and to be the source of many persistent and chronic infections [9]. The biofilm mode confers an antibiotic resistance that can be associated with difficult-to-eradicate infections such as those of medical devices [10, 11].

As far as dental caries are concerned, dental plaques, or biofilms, are formed sequentially by a number of steps. Firstly, the enamel is coated with a complex mixture of glycoproteins and glucans, bacterial debris, acidic proteins, and sialic acid; secondly, primary colonizers notably *Streptococcus sanguis* and *Actinomyces viscosus* adhere to the coated surface; thirdly, *S. mutans* adhere to the primary colonizers, albeit direct adhesion to the tooth surface has been also reported. Gradual periods of growth along with production of EPS (extracellular polymeric substance) follow until, eventually, a bulbous 3D complex is formed [12]. The production of surface proteins and acidic metabolites are cardinal to biofilm formation by this bacterium, as are the utilization of quorum sensing systems and bacteriocins production. Besides, the presence of dietary carbohydrates, particularly sucrose, accelerates this process [5]. Other variables, like the osmolarity of the medium, were noted to alter biofilm formation by other bacteria [13].

Literature has been recently accumulating to help define the genetic and molecular circuits underlying this property of the bacterium [14]. In light of this, different trials are carried out around the clock to find an agent that successfully targets it, which might culminate in a novel anti-cariogenic agent. Vitamin C (ascorbic acid) is anticipated to have such an effect, and is to be investigated in this study to understand the potency and the mechanism behind it. Vitamin C is an essential nutrient that has been found to contribute to many aspects of human health, from the very first reports on protection against scurvy to numerous contemporary evidence on its role in maintaining immunity and cardiovascular health; and cancer and chronic disease prevention, many of which were attributed to its antioxidant effects [15, 16]. In this study, of particular importance are its roles to prevent/treat dental caries and maintain oral health, which remain unchallenged despite the recent conglomeration of literature. It has been established that a correlation does exist between growth in caries activity and decreases in the salivary levels of vitamin C [17]. In fact, vitamin C was determined to be as effective as chlorhexidine in inhibiting oral microbial growth [18]. Moreover, a study investigating *Bacillus subtillis*, a model organism for biofilm development, showed an inhibitory effect of vitamin C on quorum sensing and other mechanisms underpinning biofilm development [19]. Out of serendipity and focused previous reports, we intend to exploit its effects to pave the way for establishing, along with existing evidence, the means by which we can repurpose this drug, a procedure that involves the investigation and development of existing drugs with known safety profiles for new indications in new patient populations [20]. Ultimately, the aim of this study was to determine MIC, MBC, biofilm prevention concentration (BPC), and derivative measures of vitamin C against fresh clinical isolates of *S. mutans* to evaluate its efficacy as an anti-cariogenic agent.

## Results

MIC, MBC, and BPC data were obtained from four independent experiments done in quadruplicates. There was a total of 44 samples collected, and 16 of them have been thoroughly investigated in this study.

### MIC and MBC

**Fig. 1:**
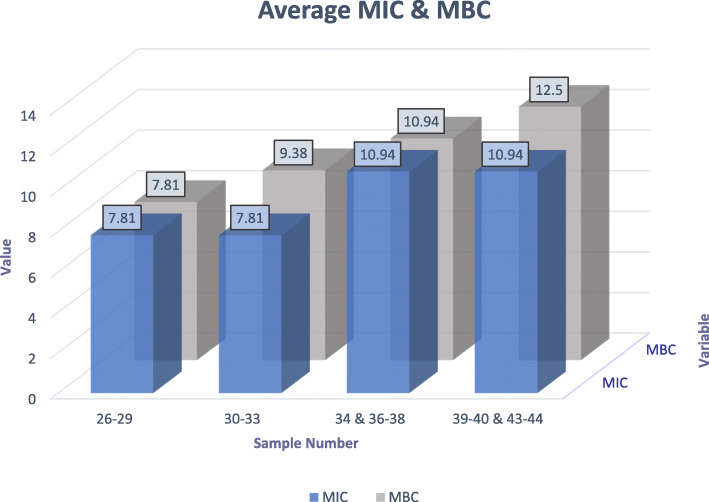
Column chart representing the average MIC and MBC values for each individual experiment done in quadruplicate

The MIC is defined as the lowest concentration of an antimicrobial agent that is bacteriostatic, or that is able to inhibit visible growth of a microorganism after overnight incubation; whereas the MBC is the lowest concentration of that agent required to kill the majority of bacterial inoculums over a fixed, somewhat extended period, as expounded by the prevention of growth of the microorganism after sub-culture to antibiotic-free media. The overall average MIC was 9.38 mg/ml of vitamin C. When the plates were sub-cultured, we found that the average MBC was 10.16 mg/ml of vitamin C. The results of each independent quadruplicate experiment are shown in Fig. 1, which were merely averaged to find out the overall result. The data provided in Additional file 1 was used to reckon these values.

### BPC and sub-BPC

**Fig. 2:**
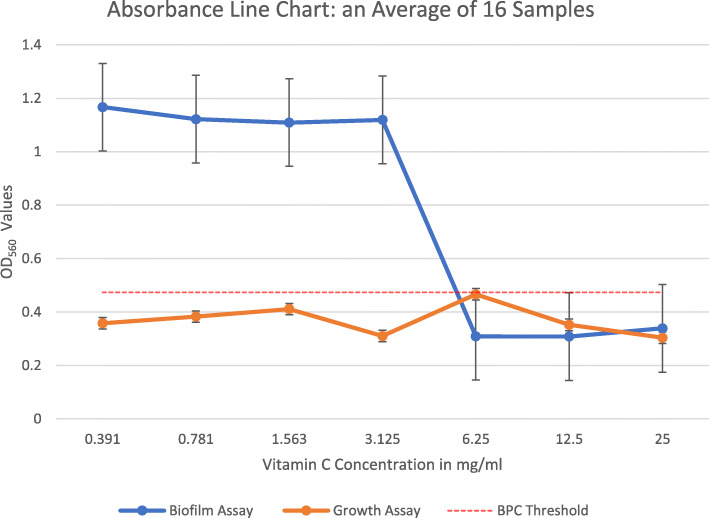
Line graph showing the relationship between absorbance at 560 nm and increasing vitamin C concentrations in the biofilm and growth assays. Note that BPC was determined by the interpolation method at the point where the blue line crossed the cutoff (0.474). Error bars represent the standard deviation of four independent experiments in quadruplicate. OD_560_: Optical density at 560 nm

The BPC is the antimicrobial concentration at which the density of a planktonic culture is sufficiently reduced to prevent biofilm formation. We found the cutoff for biofilm formation to be an absorbance value of 0.475, and, hence, we determined the BPC as the vitamin C concentration at which the absorbance was 0.474. Subsequently, BPC was interpreted using the interpolation method given the known set of data of averaged absorbances at different concentrations of vitamin C. The overall average BPC was 5.61 mg/ml of vitamin C, which can be traced on Fig. 2. The whole data set on which those values rely is provided in Additional file 2. Moreover, quantitating the second plate yielded readings that were entirely infra-threshold (Fig. 2, growth assay), which were calculated from the data in Additional file 3. We applied the two-sample (paired) t-test to compare average absorbance of our 16 samples when not exposed to vitamin C (positive controls), and that of the same strains when exposed to a supra-BPC yet infra-MBC concentration, one that definitely inhibits biofilm without exterminating the bacteria (6.25 mg/ml of vitamin C), we found a statistically significant difference between the two groups, t (15) = 4.823, *p* < 0.05 (95% CI 0.357 to 0.923). Thus, the null hypothesis was rejected.

Sub-minimal inhibitory concentration (Sub-MIC) is defined as the concentration of an antimicrobial agent that does not have an effect on bacterial growth but can alter bacterial biochemistry, thus impacting the bacterial virulence. We compared biofilm formation (absorbance values) at two sub-BPC concentrations (around ½ and ¼ BPC) and that when no vitamin C was added, under the same conditions. We found no statistically significant difference between the control group and ½ BPC group (t (15) = − 1.525, *p* > 0.05); and the control group and ¼ BPC group (t (15) = − 0.947, *p* > 0.05). Hence, the null hypothesis was not rejected.

### Kinetic study

**Fig. 3:**
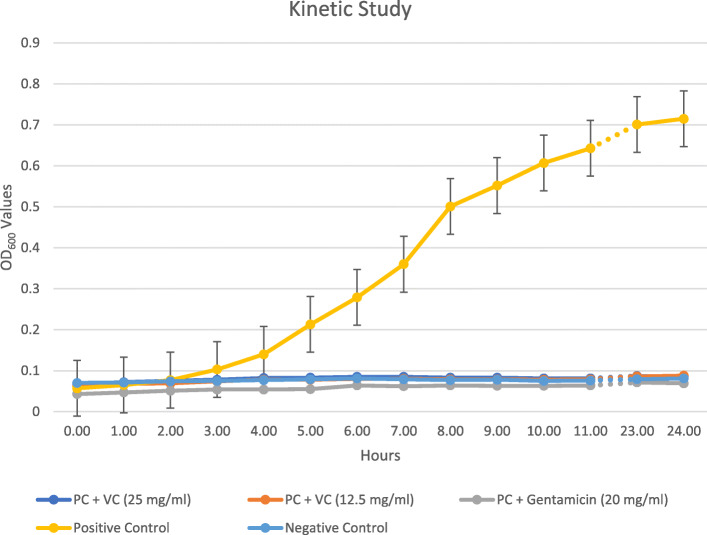
Kinetic study comparing the positive and negative controls to vitamin C and gentamicin exposed bacteria under similar conditions. Note that the absorbance of the bacteria in the positive controls increased in a time-dependent manner when compared to vitamin C- and gentamicin-exposed counterparts. PC: Positive controls. VC: Vitamin C concentration in mg/ml. OD_600_: Optical density at 600 nm

Our results showed that vitamin C precluded the growth of *S. mutans* over time when compared to the controls, and was as efficient as gentamicin in maintaining this inhibition over time. This is clearly illustrated in the line chart in Fig. 3. The data utilized in this assay is provided in Additional file 4.

### Study of synergy

Vitamin C at a concentration of 20 mg/ml produced a large zone of inhibition against the selected clinical isolates, which was three times as large as that of gentamicin. The FICI in our experiment was determined to be 0.52.

## Discussion

It is evidently discernible that an inhibitory and bactericidal effect of vitamin C exists against the *S. mutans* strains that were tested. It is generally accepted that an antimicrobial agent has a bacteriostatic activity if the MBC to MIC ratio is > 4 and is bactericidal if otherwise, although numerous factors can affect the determination of this ratio [21]. As such, the proximity of MBC to MIC variables we found denotes a fundamental correlation between them, and a bactericidal action of vitamin C against *S. mutans* growth is therefore inferred.

Moving on to the biofilm formation, we found an anti-biofilm effect of vitamin C on the same strains that were examined, based on the absorbance results that we obtained from the ELISA reader. When compared with the ELISA readings of the first plate, which were meant to assay MIC and then MBC, it was clear that biofilm did not form in the first plate. This confirms that saliva coating of the wells prior to biofilm assays and the addition of 3% sucrose to the growth medium enhances biofilm formation by *S. mutans*, as reported by Ahn et al. [14]. The anti-biofilm effect was noticed at sub-lethal concentrations, almost one-half MBC, which signifies a likely inhibitory rather than a bactericidal mechanism behind it. Besides, Vitamin C does not appear to stress the bacterium at sub-lethal doses that were smaller than BPC (sub-BPC), as these concentrations did not result in increases in biofilm formation.

It is generally accepted from multiple studies, most of which were done on gram-negative bacteria, that sub-MIC concentrations hinder bacterial virulence mechanisms [22]. In-depth, biofilm formation was also seen to be affected by sub-MIC concentrations of different antibiotics against different bacteria. This effect was inconsistent, however, with increases documented in some studies and decreases in others [23]. Nonetheless, when similar studies were applied to *S. mutans*, sub-MIC concentrations of multiple agents were shown to significantly upregulate the expression of the genes related to this process and, hence, extensively enhance biofilm formation. In fact, a study done by Dong et al. [24] showed that sub-MIC concentrations of chlorhexidine, a commonly used anti-cariogenic agent, appear to solidify and strengthen *S. mutans* biofilm. Notwithstanding those reports, the evidence is lacking in our study to support that sub-BPC concentrations of vitamin C can stimulate the bacterium to produce biofilm.

We recognize that, although we followed the guidelines that were set forth by Thieme et al. [25] in testing biofilm susceptibility, our method might have contained weaknesses sufficient to slightly alter our measurements. Crystal violet is a simple yet reliable method for total biomass quantitation, and remains the most commonly used quantitation technique in microtiter plate assays despite its limitations; a combination of different methods, nevertheless, is the ultimate reliable method for biofilm studies [26, 27]. We must acknowledge, however, a tangible deficit of proper methodology among different anti-biofilm component studies, resulting in inconsistency and discrepancies between laboratory and clinical evidence. Hence, it is necessary that standardized methods with accurate and precise definitions of biofilm susceptibility endpoint parameters are put forth in the coming years. We also admit that by targeting *S. mutans* in our attempt to reveal an anti-cariogenic effect of vitamin C, we might be omitting the other contributing factors to cariogenesis, especially that the ecological plaque hypothesis is now the more favored hypothesis. Nonetheless, we cannot divest *S. mutans* of its prominent role in cariogenesis that remains valid even with improved isolation techniques of the plaque microflora. Thus, it is entrenched that negating *S. mutans* functionality in dental plaques would not completely eradicate dental carries but would significantly diminish its incidence [2].

The kinetic study is, in essence, a study that helps in providing a mechanistic understanding of how antimicrobial agents influence microbial populations and how different concentrations alter their activity. Otherwise stated, it aims to evaluate the activity of the antibiotic against a sensitive test microorganism. The kinetics of vitamin C at supra-MBC concentrations show a stable, maintained inhibitory effect over a period of 24 h. The action started instantly after its administration, and no bacterial growth was observed throughout the period. When it was combined with gentamicin, no synergistic effects were seen at different concentrations. Although previously conducted studies with a similar methodology evinced a synergistic activity of vitamin C with streptomycin and kanamycin against multi-resistant *Pseudomonas aeruginosa* strains [28], the FICI we found, according to the mentioned definitions in the methodology, roughly indicates that no interaction exists between vitamin C and gentamicin against the *S. mutans* strains tested.

A particular area of focus in future endeavors should be to determine the optimal dose to obtain such a beneficial effect of vitamin C without unwanted adverse effects. It is generally accepted that the recommended daily allowance (RDA) of vitamin C is 90 mg/ml for males and 75 mg/ml for females, with a tolerable upper level (TUL) of 2 g that was set based on the gastrointestinal upset that accompanies excessive doses [29]. As far as the anti-cariogenic effect is concerned, there has been a case report of a demineralizing effect in the form of localized tooth wear secondary to vitamin C-induced acid erosion. It has been shown that tablets containing 60 mg or more of vitamin C will cause a reduction in salivary pH when used for a prolonged period of time, albeit a host of other factors appear to play a role [30]. The same effect was also reported by a study done on children with dental erosion [31]. Therefore, it is of utmost importance that in vivo studies are carried out before determining the optimal dose, especially that an inoculum effect, wherein discrepancies are created between laboratory and clinical tests due to the dependency of doses on bacterial density, and a multitude of other factors like inherent assay variation make laboratory tests less reproducible [32, 33]. This can be a particular concern if an idea of a vitamin C-containing mouth wash or toothpaste was to be implemented for dental caries patients.

## Conclusion

In conclusion, our results point towards a neglected anti-biofilm and, therefore, potentially anti-cariogenic effect of vitamin C on *S. mutans*. We can deduce from our results that the BPC we found was essentially a sub-lethal concentration at which this effect begins preventing biofilm development, through a mechanism that is yet to be rigorously scrutinized. To the best of our knowledge, this is the first report that employs the BPC and its derivate measures in studying the effect of vitamin C on this important property of *S. mutans*, in a set up that is finest considering our laboratory capacity. This study embodies a paradigm that adds on to the pool of literature in an attempt to repurpose vitamin C as a novel cariogenic agent. Our results are a piece of mere ancillary evidence that augments previous reports, through which we intend to stimulate the initiation of trials of a higher level of evidence to increase our know-how of this topic.

## Methods

### Samples collection

The consenting patient with caries/poor oral health, as identified by the dentist in RAK College of Dental Sciences (RAKCODS) by having at least two active dental caries and a fair or poor oral health, was asked to chew an orthodontic elastic for 2 min. This was placed in his mouth by the principal investigator or one of the co-investigators. It was necessary that the patient was 20–55 years old and had not eaten or drunk for 2 h prior; it was also required that they were previously and currently clear of diabetes, cancer, and systemic infections. Similarly, a healthy volunteer, as reported by the dentist, underwent the same procedure and a healthy sample was procured from him. The participant was then asked to spit into a chilled sterile container provided. Afterward, the stimulated whole saliva samples were packaged with care to not allow contamination, then delivered within 30–60 min to the laboratory.

We opted to collect stimulated saliva samples because they exhibit a more even and true representation of the oral ecosystem [34]. Although the genus *Streptococcus* is by far the most abundant in unstimulated saliva samples, it appears in higher quantities yet in lower proportions in stimulated saliva samples, probably because of plaque removal during chewing of paraffin in stimulated saliva sample collection.

### Samples processing

The unhealthy saliva samples (from patients with caries/poor oral health) were cultured on TYCSB (tryptone yeast extract cystine with sucrose and bacitracin) agar to allow the growth of *S. mutans* from those samples. The culture plates were then kept in the incubator in a candle jar (5% CO_2_ atmosphere) for 48 h at 37 °C. Afterward, they were sub-cultured on BHI (brain heart infusion broth) in test tubes. These tubes were covered with parafilm and kept in the incubator in a candle jar overnight at 37 °C. TYCSB agar was chosen in this study because it was determined to be the most sensitive and selective media for culture of *S. mutans* for laboratory and clinical studies, as the recovery of the laboratory *S. mutans* strain was highest in TYCSB agar when compared to other commercially available selective media; and ratios of mutans to non-mutans bacteria were also highest in TYCSB [35].

Meanwhile, saliva samples from healthy volunteers were centrifuged after collection at 4000×g for 10 min to remove cellular debris. They were then subjected to filter sterilization through a 0.22-μm acrodisc filter. The clarified saliva at the bottom was then stored in the freezer at 0 °C until they were used to coat the wells of the microtiter plate.

### Biofilm and growth assays preparation

**Fig. 4:**
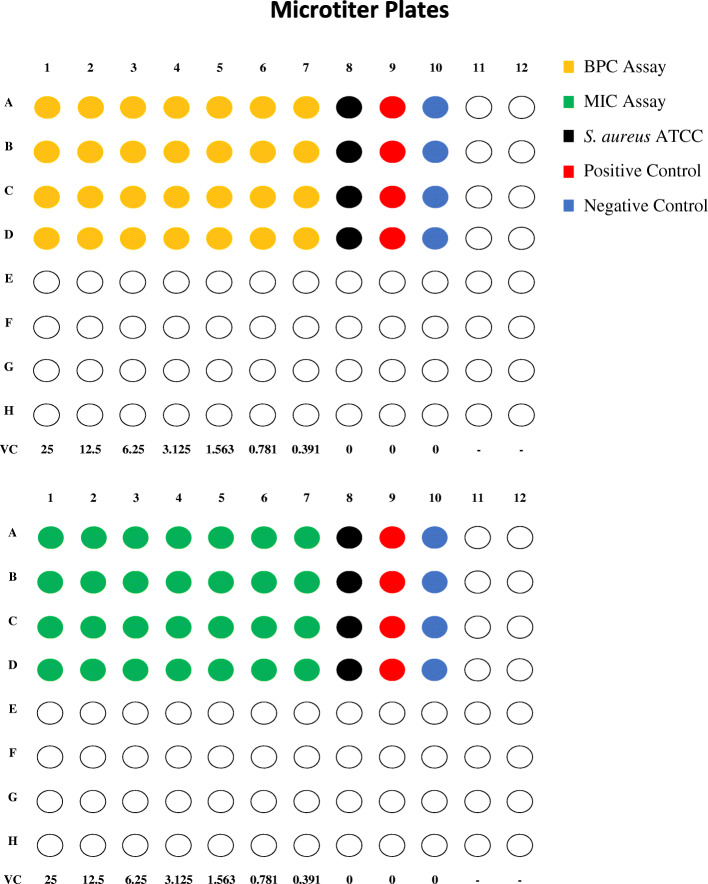
Graphical representation of the first (above) and the second (below) microtiter plates. Four samples were processed at a time. Each row in the plates represents a saliva sample, such that different properties were assayed using the same four samples in rows A**–**D of the first plate and A**–**D of the second plate. VC: Vitamin C concentration in mg/ml

Biofilm formation by *S. mutans* was assayed using the component crystal violet staining method described by Ahn et al. [14] with some modifications. This method, originally described by O’Toole and Kolter in 1998 to identify biofilm-deficient mutants [13], remains the most frequently used quantitation technique in microplate assays, despite its limitations and the development of more advanced methods [26]. The assays were done using polystyrene 96-well flat-bottom microtiter plates in quadruplicates, in which four different samples from patients with caries were processed at a time. Two microtiter plates were used for each set of samples (Fig. 4). The first plate was used to assay the inhibitory effect of vitamin C on biofilm formation by *S. mutans*; the second plate was used to assay the inhibitory effect of vitamin C on bacterial growth (minimum inhibitory concentration or MIC). Accordingly, conditions were made more favorable for biofilm formation in the former plate, as exemplified by saliva-coating of the wells and addition of 3% sucrose to the broth, and less favorable in the latter by eliminating those two factors. Filtered salivary preparations from the healthy volunteer were used to coat the wells of the first microtiter plate, before adding the cell suspensions. This method is thought to promote bacterial adherence and, therefore, biofilm formation by providing binding receptors to bacterial proteins. To achieve this, each well was conditioned with 100 μl of filtered saliva. The plate was incubated at 37 °C for 2 h with gentle shaking and then washed three times with PBS (phosphate-buffered saline). The wells were then allowed to air-dry for 30 min.

### Serial dilutions of vitamin C

The plates were processed as follows: 200 μL of vitamin C at a concentration of 25 mg/mL was added to the wells of the first column; 100 μL of broth (BHI) was also added to the next nine wells along the same row. Serial two-fold dilutions were then carried out from the first to seventh wells, such that vitamin C concentration was 25 mg/ml in the first well and 0.391 mg/ml in the seventh well. As for the eighth well, it was kept as a control for biofilm formation wherein *Staphylococcus aureus* ATCC (American type culture collection) 25,923, a bacterium with a known capacity of biofilm formation, was grown. Besides, the ninth and tenth wells were kept as positive and negative controls, respectively. Ten μL of *S. mutans* subculture from the respective sample and cultured *Staphylococcus aureus* ATCC at 0.5 McFarland standard were added into wells one to nine (except eight) and eight, respectively. Ultimately, each well (except the tenth) contained 110 μL and each saliva sample was represented by a row on the microtiter plate; with columns one through seven being serial dilutions of vitamin C onto cell suspensions, column eight being the control of biofilm formation, nine being the positive control, and 10 being the negative control (Fig. 4). The microtiter plates were then placed in the incubator at 37 °C in the candle jar for 48 h.

### Growth assays

The growth on the second microtiter plate was interpreted visually to determine the minimum inhibitory concentration (MIC) using the aforesaid microtiter plate broth dilution or microdilution method, wherein the vitamin C concentration in the last well from columns one to seven in each row visually lacking growth was identified as MIC. The overall average MIC was calculated by averaging the MICs of all the samples we tested, each representing a row on the second microtiter plate (Fig. 4). Following this, on a single TYCSB or blood agar, each row with its first 10 wells was sub-cultured by transferring 10 μL into separate zones of the agar plate to determine the minimum bactericidal concentration (MBC). The agar plate was then kept in the incubator at 37 °C in a candle jar for 24 h. Afterward, MBC was visually interpreted as the vitamin C concentration in the last zone of the agar not showing growth. The overall average MBC was calculated by averaging the MBCs of all the samples we tested in our four quadruplicate experiments. A similar setup was used by Moradian et al. [36] to determine the MIC and MBC of certain compounds against *S. mutans*.

### Biofilm assay

On the same day, the biofilm formation assay was performed for both plates by the crystal violet staining method. Firstly, the contents of the well, comprising planktonic or free-floating bacteria, were removed with care; leaving behind the growth at the bottom of the well. Each well was then gently washed twice with 200 μL of sterile distilled water and dried for 10 min. Fifty μL of 0.1% crystal violet was then added to the wells, and the plates were kept for 15 min at room temperature to stain the remaining adherent bacteria. After the wells were rinsed twice with 200 μl of distilled water, the bound dye was extracted from the stained cells using 200 μl of 99% ethanol, which was left for 5 min. The biofilm was then quantitated by measuring the absorbance of the solution at 560 nm in the GloMax microplate ELISA reader (GM3500, Promega, Madison, WI, USA). The readings from the first plate were then used to determine the BPC (see next paragraph), whereas those from the second plate were merely used to confirm that the conditions we placed this plate in were not favorable for biofilm formation. In other words, biofilm is not expected to form in the second plate, and, therefore, the readings were anticipated to be entirely infra-threshold.

Multiple susceptibility endpoint parameters have been used to assess biofilms in recent years, including the minimal biofilm eradication concentration (MBEC), the minimal biofilm inhibitory concentration (MBIC), the biofilm bactericidal concentration (BBC), and the biofilm prevention concentration (BPC). Except for BPC, all other parameters require the analysis of the activity of a certain antimicrobial agent on established biofilms, and then to compare it with post-treatment readings to infer the antibiofilm activity. If the number of viable cells remained static, an inhibitory effect is inferred (MBIC); whereas if the number of viable cells decreases, an eradication effect is inferred (MBEC) [25]. In contrast, the BPC determines the antimicrobial concentration at which the density of a planktonic culture is sufficiently reduced to prevent biofilm formation. Otherwise stated, it assays an inhibitory effect of antimicrobials on biofilm formation, with the bacterial inoculation and antimicrobial exposure occurring simultaneously rather than exposing an established biofilm to antimicrobials [37].

In this study, the inoculation of *S. mutans* clinical isolates and vitamin C exposure occurred simultaneously, without allowing pre-treatment biofilm to form. Hence, we assessed the BPC of vitamin C by measuring the absorbance of stained post-treatment biofilms at increasing concentrations of vitamin C, by the aforementioned method. We determined the threshold of BPC using the *S. aureus* ATCC that we included in our study as the positive control for biofilm formation. Consequently, we averaged the absorbance of *S. aureus* biofilm, from the four quadruplicate experiments. Afterward, this value was used as a cutoff for determining BPC, wherein the vitamin C concentration at and above this value prevented biofilm formation by the clinical isolates; as compared with exposure to lesser doses of vitamin C, in which biofilm formation occurred freely. Since the BPC value we determined was in between the serial dilutions that we have done (see results), we calculated it using the interpolation method. This method uses a discrete set of known data points, representing the absorbance values at the six 2-fold serial dilutions of vitamin C, to construct a new data point within their range, which was the BPC in our case.

### Statistical analysis

To establish the statistical significance of our findings, we compared two sets of results in a dependent observation using the two-sample (paired) t-test and estimated the range of difference between their means by measuring the confidence interval. We used SPSS software (statistical package for the social sciences, version 26, International Business Machines Corporation, Armonk, NY, USA) to carry out the statistical tests, all of which were two-sample t-tests, and the significance level was determined at *p* < 0.05. All data sets were determined to be normally distributed by the Shapiro-Wilk test. First, we compared the average absorbance value of the samples when not exposed to vitamin C (positive controls), and that of the same strains when exposed to a supra-BPC yet infra-MBC (6.25 mg/ml) concentration of vitamin C. We also compared the average absorbance value of the positive controls with that of two sub-BPC concentrations (around ½ and ¼ BPC).

### Kinetic study

The kinetic study is essentially a turbidimetric assay that allows the evaluation of microbial growth throughout incubation time. Selected samples were used in this study. Overnight cultured samples were conditioned on a microtiter plate with two supra-MBC concentrations and their absorbance was measured over a period of 24 h. Six wells were used for each sample: the first being the positive control; the second and third containing bacteria/broth with vitamin C at 25 mg/ml and 12.5 mg/ml, respectively; the fourth and fifth containing bacteria/broth with gentamicin at 20 mg/ml and 10 mg/ml, respectively; and the sixth being the negative control. The plate was appropriately covered and incubated at 37 °C and 5% CO_2_ between the readings. The results were then plotted on a graph of time against absorbance. In this study, it was expected that if growth occurs, the turbidity will increase and, hence, the absorbance values will gradually grow over time. On the contrary, if the agent, such as vitamin C or gentamicin, interacted with *S. mutans* sufficiently to alter its growth kinetics; an altered response, as epitomized by the turbidity and hence the absorbance after exposure, will be seen [32, 38]. Although this study uses the absorbance to assess the turbidity, it is not a measure of biofilm formation as implemented previously in this study.

### Comparison with gentamicin and study of synergy

Selected samples were used to gauge the difference between the inhibitory capacity of vitamin C and gentamicin against *S. mutans*, and to study the presence of any synergistic effects between them. Samples were cultured on blood agar and the zones of inhibition of each were compared when alone and when combined together. As for studying any possible synergy or antagonism between them, the fractional inhibitory concentration index (FICI) was used. It can be calculated by the following equation: (MIC_A_/MIC_AB_) + (MIC_B_/MIC_AB_), where A and B are the two drugs tested alone, or in combination (AB). The MIC for both vitamin C and gentamicin was measured using the same strains under the same conditions. FICI was interpreted according to standard definitions, wherein a FICI of ≤0.5 was determined as a synergistic effect, a FICI of > 4.0 was determined as an antagonistic effect, and a FICI between 0.51 and 3.99 was determined as no interaction [39, 40].

## Supplementary information

**Additional file 1.** Microsoft excel worksheet. MBC and MIC Assays. This worksheet provides the data from which the average MBC and MIC were calculated.

**Additional file 2.** Microsoft excel worksheet. Biofilm Assay. This worksheet provides the data of the readings of the first plate, which is plotted in Fig. 2 as “Biofilm Assay”. This dataset was used to calculate the BPC of vitamin C. Note that the cells were conditionally formatted using a color scale, wherein the values that surpassed the BPC absorbance value were colored in red, those less than BPC were colored in green, and those at the borderline were colored in yellow.

**Additional file 3.** Microsoft excel worksheet. Growth Assay. This worksheet provides the data of the readings of the second plate, which is plotted in Fig. 2 as “Growth Assay”. Note again that the cells were conditionally formatted using a color scale, wherein the values that surpassed the BPC absorbance value were colored in red, those less than BPC were colored in green, and those at the borderline were colored in yellow.

**Additional file 4.** Microsoft excel worksheet. Kinetic Study. This worksheet provides all the data that was necessary to construct Fig. 3. Here the cells were conditionally formatted using a color scale but without a cutoff. The cells with the highest values were colored in red, the ones with the lowest values were colored in green, and the ones in between were colored in yellow.

## Data Availability

Most of the datasets generated or analyzed during this study, based on which conclusions were derived, are included in this published article as additional files.

## Abbreviations

EPS: Extracellular polymeric substance
MIC: Minimum inhibitory concentration
MBC: Minimum bactericidal concentration
BPC: Biofilm prevention concentration
MBIC: Minimum biofilm inhibitory concentration
MBEC: Minimum biofilm eradication concentration
BBC: Biofilm bactericidal concentration
RAKCODS: RAK college of dental sciences
TYCSB: Tryptone yeast extract cystine with sucrose and bacitracin agar
BHI: Brain heart infusion broth
VC: Vitamin C
PBS: Phosphate-buffered saline
ATCC: American type culture collection
ELISA: Enzyme-linked immunosorbent assay
FICI: Fractional inhibitory concentration index
OD: Optical density
SPSS: Statistical package for the social sciences
CI: Confidence interval
PC: Positive control
RDA: Recommended daily allowance
TUL: Tolerable upper level
